# Associations of actigraphic sleep and circadian rest/activity rhythms with cognition in the early phase of Alzheimer’s disease

**DOI:** 10.1093/sleepadvances/zpab007

**Published:** 2021-04-27

**Authors:** Alfonso Alfini, Marilyn Albert, Andreia V Faria, Anja Soldan, Corinne Pettigrew, Sarah Wanigatunga, Vadim Zipunnikov, Adam P Spira

**Affiliations:** 1 Department of Neurology, Johns Hopkins School of Medicine, Baltimore, MD; 2 Department of Radiology and Radiological Science, Johns Hopkins School of Medicine, Baltimore, MD; 3 Department of Biostatistics, Johns Hopkins Bloomberg School of Public Health, Baltimore, MD; 4 Department of Mental Health, Johns Hopkins Bloomberg School of Public Health, Baltimore, MD

**Keywords:** actigraphy, sleep, circadian rhythms, cognition, Alzheimer’s disease

## Abstract

**Study Objectives:**

To compare sleep and circadian rest/activity rhythms (RARs), quantified by standard and novel actigraphic metrics, between controls and participants with mild cognitive impairment (MCI), and to examine the cross-sectional relationships between these measures and cognition.

**Methods:**

Actigraphy data were collected in 179 older individuals (mean age = 72.6 years) with normal cognition (*n* = 153) and MCI (*n* = 26). Sleep parameters (e.g. sleep efficiency), and standard nonparametric RARs (e.g. interdaily stability) were generated. Functional principal component analysis (fPCA) was used to generate three novel RAR metrics (fPC1, fPC2, and fPC3). Cognitive composite scores reflecting episodic memory and executive function were derived using factor analysis. Regression models compared sleep and RAR parameters between diagnostic groups and their association with cognitive performance.

**Results:**

Compared to controls, the MCI group exhibited lower levels of the standard RAR parameter: relative amplitude and fPC3—a novel RAR whereby lower scores reflected a lower rhythm peak, as well as greater nighttime activity and less activity in the morning. Across groups, several standard RAR parameters (e.g. interdaily stability) and fPC3 were associated with better episodic memory and executive function performance. Additionally, several standard RAR measures (e.g. relative amplitude) and the novel RAR measure fPC1 (reflecting the total volume of activity and rhythm strength) were associated with better executive function performance.

**Conclusions:**

Individuals with MCI have altered circadian RARs compared to controls, including the novel RAR metric fPC3, reflecting greater nighttime activity and less activity in the morning compared to mean values. Additionally, these measures are significantly associated with cognitive performance.

**Statement of Significance**

Sleep and rest/activity rhythms (RARs) are altered in individuals with Alzheimer’s disease (AD) dementia. Much remains unknown about alterations that may be present in the earlier phases of AD and their association with cognition. Most studies in this domain have used subjective sleep assessments or standard actigraphic methods to generate sleep and circadian RAR measures. To improve our understanding of the associations of sleep and circadian rhythms with cognitive performance in early AD, we compared standard sleep and circadian RARs, as well as novel circadian RARs (based on functional principal component analysis), between controls and those with mild cognitive impairment, and examined their cross-sectional relationships with episodic memory and executive function performance in the combined sample.

## Introduction

Evidence indicates that individuals with Alzheimer’s disease (AD) dementia have altered sleep, including longer and more frequent awakenings at night and naps during the day [1]. AD dementia patients also exhibit altered actigraphic circadian rest/activity rhythms (RARs), including lower amplitude, less consistent, and more fragmented rhythms, and the timing of the rhythm peak is earlier in the day than in cognitively normal individuals [2–4]. Similarly, individuals with mild cognitive impairment (MCI) spend more time awake after sleep onset, and exhibit an earlier onset of melatonin secretion, suggesting a more advanced circadian phase compared to normal controls [5, 6]. Changes in sleep and circadian rhythms are also common in normal aging, with cognitively normal older individuals often exhibiting a shortened sleep duration, less consolidated sleep, and blunted circadian rhythms compared to their younger peers [7].

Although several lines of evidence indicate that poor sleep and altered circadian rhythms are present among individuals along the AD continuum [8], much remains unknown about the nature of such changes in the early phases of AD and their associations with cognitive performance. Few investigations have examined these relationships, and of these, most have employed standard actigraphic approaches to generate sleep and circadian parameters. For instance, several large cohort studies comprised of community-dwelling older adults with varying degrees of cognitive ability—including participants with probable dementia and MCI—have demonstrated cross-sectional associations of actigraphic indices of poor sleep and altered RARs with poorer cognitive performance. Findings from a study of older men revealed that greater sleep disturbance and longer sleep duration were associated with worse executive function and global cognitive performance [9]. Results from a similar study of older women demonstrated that longer sleep duration was associated with poorer global cognition and that greater sleep disturbance was linked with lower verbal fluency and working memory performance [10]. Fragmented RARs in non-demented persons have also been related to slower processing speed, worse working memory, and poorer visuospatial performance [11].

While prior studies demonstrate that disrupted sleep and altered circadian RARs are associated with cognitive performance, they provide limited information about the dynamics of the sleep and circadian patterns that characterize individuals in the early phases of AD. In the current study, we compared sleep and circadian RARs, using standard and novel actigraphic measures in cognitively normal adults and individuals with MCI, and examined the cross-sectional associations of these metrics with cognitive performance among all participants. Based on the research described above, we hypothesized that MCI subjects would exhibit more fragmented sleep and lower amplitude, less consistent RARs than normal controls, and that greater sleep fragmentation and more disrupted RARs would be associated with poorer episodic memory and executive function performance. Additionally, because standard RARs focus on select aspects of circadian rhythms, they lack information regarding the rhythms’ shape and oscillatory pattern over the 24-hour period. In contrast, the novel RAR measures presented here provide information about shifts in rest/activity patterns over a 24-hour cycle, and thus may yield more comprehensive and nuanced information about alterations in circadian profiles. Thus, we hypothesized that novel circadian RAR parameters would yield integrated information about pattern differences that would provide additional insights into the changes that occur during the early phases of AD, and that such measures would also be associated with cognitive performance.

## Methods

### Study design and participant selection

Analyses were conducted using data from the Biomarkers for Older Controls at Risk of Dementia (BIOCARD) study, which is an ongoing prospective cohort study. The BIOCARD study was initiated in 1995 at the National Institutes of Health (NIH) and was designed to enroll and follow cognitively normal individuals with the goal of identifying variables that predict subsequent development of mild to moderate symptoms of AD. At baseline, after completing a comprehensive evaluation and providing written informed consent, a total of 349 participants were enrolled in the BIOCARD study. By design, about 75% of the BIOCARD cohort had a first-degree relative with AD-type dementia. From 1995 to 2005, participants received annual clinical and cognitive assessments. Additionally, cerebral spinal fluid (CSF) and magnetic resonance imaging (MRI) scans were collected approximately every other year. In 2005 the study was discontinued for administrative reasons. In 2009, an investigative team at the Johns Hopkins School of Medicine was funded to re-establish the study and resume clinical and neuropsychological evaluations. In 2015, MRI scans and CSF acquisition were reinstated, and amyloid imaging began. In 2016, actigraphy data collection was initiated. Additional details related to participant recruitment, clinical evaluation, and cognitive assessments have been described elsewhere [12]. The BIOCARD study was approved by the Johns Hopkins University Institutional Review Board.

The current report examines the cross-sectional associations of sleep and circadian RARs with cognitive performance among 179 BIOCARD study participants whose data were collected between 2016 and 2019.

### Clinical and cognitive assessments

BIOCARD participants receive clinical evaluations and cognitive assessments during their annual visits, which include a semi-structured interview, based on the Clinical Dementia Rating (CDR) scale [13], and a battery of neuropsychological tests. Consensus diagnoses are generated annually for each participant by the BIOCARD Clinical Core staff at the Johns Hopkins School of Medicine. This clinical team uses procedures comparable to those established by the National Institutes on Aging (NIA) Alzheimer’s Disease Centers program. A syndromic diagnosis is generated first, which is based on the following information: (1) the clinical data describing the medical, neurological, and psychiatric status of the individual; (2) reports of cognitive changes by the individual and collateral sources; and (3) diminished cognitive performance over time, based on review of the longitudinal data (and relative to published age-matched norms). The syndromic diagnostic categories were: (1) cognitively normal (referred to as “normal” throughout the manuscript), (2) MCI, (3) impaired not MCI, and (4) dementia. If an individual is presumed to be cognitively impaired, a decision regarding the probable etiology of the syndrome is determined based on the clinical information collected at each visit, and the individual’s medical history, if warranted. Multiple etiologies for a single participant are possible. The consensus diagnostic protocol was guided by the recommendations outlined in the NIA/Alzheimer’s Association working group reports for the diagnosis of MCI [14] and dementia due to AD [15]. The diagnosis of “Impaired not MCI” was typically given if there was contrasting information from the CDR interview and the cognitive test scores (i.e. the subject or collateral source reported concerns about cognitive changes in daily life, but the cognitive testing did not show changes, or vice versa). Because participants with a diagnosis of “Impaired not MCI” (*n* = 31) do not meet criteria for MCI, they were included among the group of normals, consistent with prior publications (see Albert et al. [12] for additional details).

Twelve test scores from the neuropsychological battery [12] were used to create cognitive composite scores. Factor analytic techniques were used to create cognitive composite scores reflective of four fundamental cognitive domains. Raw scores from each cognitive task were standardized, using a z-transformation. Standardized scores were weighted by their own factor loadings and summed within each domain to generate a composite score for: (1) episodic memory; (2) executive function; (3) language; and (4) visuospatial ability, which have been used in prior publications (e.g. Soldan et al. [16]). The cognitive composite scores examined in the present study included episodic memory and executive function, which were analyzed as continuous variables.

### Actigraphy data acquisition and quantification of standard sleep parameters

Following their in-person visit, participants were asked to wear an actigraph (Actiwatch-2, Philips Respironics, Bend, OR) on their non-dominant wrist for seven consecutive days. During data collection, participants were instructed to press an event-marker button on the actigraph at “lights out” when they intended to sleep, and again upon awakening when they no longer intended to sleep. Upon awakening, participants were instructed to report in their sleep diary the times at which they intended to sleep (“lights out”) and arise from bed to start the day. Participants were also instructed to report times of any removal of the actigraph, travel across time zones, and naps. Actigraphy data were acquired continuously in 30-second epochs and a minimum of three 24-hour intervals of usable data were required for inclusion in the analyses. Actigraphy data were deemed invalid during participant-reported periods of travel across time zones, illness, non-wear time, and device malfunction. The raw actigraphy data were exported using Actiware Software (v. 6.0, Philips Respironics, Bend, OR) and scored by two independent raters using information provided in the sleep diary, with no knowledge of the clinical or cognitive status of the participants.

Four standard nighttime sleep parameters were extracted from the validated actigraphy data, employing a widely used algorithm [17]: (1) total sleep time (TST; number of minutes slept while in bed); (2) sleep efficiency (SE; proportion of time in bed asleep, %); (3) wake after sleep onset (WASO; number of minutes awake after the initial sleep bout); and (4) average wake bout length (WBL; number of minutes awake divided by the number of wake bouts). These standard actigraphic sleep parameters were averaged across nights and analyzed as continuous variables.

### Epoch-by-epoch actigraphy data pre-processing for quantification of circadian metrics

In preparation for the generation of the circadian metrics, the raw epoch-by-epoch actigraphy data (i.e. total activity counts per 30-second epoch) were pre-processed via R Software (v. 3.6.1, R Core Team, 2019) using the following procedures: (1) 24-hour intervals with more than 72 minutes (i.e. > 5% of the 24-hour interval) of missing data were discarded; remaining missing data that did not exceed this threshold were imputed using the average minute-level values across intervals; (2) The raw actigraphy data were summed over consecutive (30-second) epochs and log-transformed, producing within-subject minute-level data for each 24-hour interval; (3) Minute-level data were then binned on an hourly basis and averaged across all 24-hour intervals to generate a time-series of 24 data points for each participant [18–20].

### Quantification of standard circadian RAR parameters

Standard nonparametric circadian RAR parameters were generated from the processed time-series data, using the ActCR package in R [21]. This standard nonparametric approach was used to calculate three measures: (1) intradaily variability (IV; within-day rhythm fragmentation), (2) interdaily stability (IS; between-day rhythm consistency), and (3) relative amplitude (RA; rhythm height and overall measure of rhythm strength) [4].

Though not the primary focus of these analyses, supplemental standard parametric circadian (cosinor) metrics were also generated. These included three measures: (1) amplitude (difference between the rhythm nadir and rhythm peak); (2) midline estimating statistic of the rhythm (MESOR; average rhythm height over the 24-hour interval); and (3) acrophase (timing of the rhythm peak) [22, 23] (see Supplementary Materials for details).

### Novel circadian RAR measures

In addition to the standard sleep and circadian RAR measures described above, functional principal component analysis (fPCA) was used to generate several novel circadian RAR parameters, using the refund [24] and mgcv packages for R [25]. To generate these data, the pre-processed time-series data were decomposed and nonparametrically modeled to generate the most dominant circadian patterns across the sample.

### APOE genotyping and coding

APOE genotyping was conducted using restriction endonuclease digestion of polymerase chain reaction amplified genomic DNA (Athena Diagnostic, Worcester, MA). APOE genotypes were dichotomized to reflect ε 4carrier status. ε 4carriers (individuals with at least one ε 4allele) were coded as 1, and non-carriers (individuals with no ε 4 allele) were coded as 0.

### Statistical analysis

Group differences (Normal vs. MCI) were examined using independent samples *t*-tests or Wilcoxon rank-sum tests for continuous variables. Chi-square or Fisher’s exact test were used for categorical variables. Correlations between the standard and novel sleep and circadian RAR parameters were calculated using the Pearson product moment correlation test.

Primary analyses were conducted using multivariable linear regression. All models were adjusted for age, gender, years of education, and APOE ε 4 status. First, the associations of diagnostic status with each sleep and circadian RAR parameter were tested in separate models, with diagnosis (Normal = 0, MCI = 1) as the predictor and one sleep or circadian RAR parameter as the outcome variable. Second, the associations of sleep and circadian RAR parameters with each cognitive composite measure were tested in separate models, with one sleep or circadian RAR parameter as the predictor and one cognitive composite measure as the outcome variable. Two sets of sensitivity analyses were completed. First, we examined whether any significant result differed by APOE status. To test this, we added an interaction term (e.g. SE × APOE) to each of the models above that yielded a significant finding. Second, we examined whether significant results differed when excluding individuals with a diagnosis of “Impaired Not MCI” by repeating all analyses excluding these individuals.

Our primary analyses examining sleep-related differences used the Šidák test [26] to control the type 1 error rate (i.e. number of tests = 12), in which a more stringent statistical significance threshold was enforced (α = 0.004). Analyses comparing circadian RARs—quantified by standard and novel actigraphic indices—between Normals and individuals with MCI, and analyses examining the links between these measures and cognition were not corrected for multiple comparison. Before model fitting, all continuous variables were standardized. These analyses were conducted using Stata software (v. 15.1; StataCorp, College Station, TX).

## Results

A total of 179 BIOCARD participants had at least three 24-hour intervals of valid actigraphy data and at least one cognitive composite measure. Participants with fewer than three valid 24-hour intervals of actigraphy data (*n* = 6) were excluded. On average, actigraphy and cognitive data were obtained 5.9±7.4 months apart.

The demographic and genetic characteristics of the entire sample (*n* = 179), as well as the two diagnostic groups (Normal = 153, MCI = 26) are shown in Table 1. The MCI subjects were older and had lower episodic memory and executive function composite scores, but there were no other demographic or genetic differences between the groups. Most of the MCI subjects (92.3%) had an etiologic diagnosis of possible or probable AD, as determined by the consensus review panel (Table 2).

**Table 1.** Participant characteristics by clinical diagnosis

Characteristic	Total sample (*n* = 179)	Normal (*n* = 153)	MCI (*n* = 26)	*p* (effect size)
Age, mean±SD, years	72.6±8.4	71.8±8.3	77.3±7.9	**0.002 (0.67)**
Female, *n* (%)	116 (64.8)	103 (67.3)	13 (50)	0.087 (0.36)
Education, mean±SD, years	17.3±2.2	17.3±2.3	17.5±2.1	0.745 (0.08)
White, *n* (%)	174 (97.2)	149 (97.4)	25 (96.2)	0.548 (0.09)
APOE ε4 Carrier, n (%)	57 (31.8)	47 (30.7)	10 (38.5)	0.433 (0.17)
Episodic Memory, mean±SD, score	1.4±1.7	1.8±1.4	-0.5±1.9	**<0.001 (1.58)**
Executive Function, mean±SD, score	0.2±1.3	0.4±1.3	-1.2±0.9	**<0.001 (1.35)**
TST, mean±SD, minutes	411.2±55.5	413.1±52.7	400.4±70.3	0.103 (0.07)
SE, mean±SD, %	85.3±6.7	85.7±6.9	83.1±5.6	**0.019 (0.05)**
WASO, mean±SD, minutes	39.9±17.3	39.3±17.1	42.9±18.6	0.219 (0.02)
WBL, mean±SD, minutes	1.3±0.4	1.3±0.4	1.3±0.5	0.868 (0.03)
Standard RAR IS, mean±SD, score	0.8±0.1	0.8±0.1	0.8±0.1	0.139 (0.01)
Standard RAR IV, mean±SD, score	0.5± 0.2	0.5±0.2	0.6±0.2	0.198 (0.05)
Standard RAR RA, mean±SD, score	0.8±0.1	0.8±0.1	0.7±0.1	**<0.001 (0.10)**
fPC1, mean±SD, score	0.0±154.9	5.4±157.1	-31.9±139.6	0.820 (0.09)
fPC2, mean±SD, score	0.0±138.3	-4.6±136.2	27.3±150.2	0.344 (0.03)
fPC3, mean±SD, score	0.0±78.2	4.2±76.7	-24.6±83.5	**0.036 (0.05)**
Actigraphy Days, mean±SD, days	5.6±0.9	5.6±0.8	5.4±1.0	0.142 (1.35)

TST, total sleep time; SE, sleep efficiency; WASO, wake after sleep onset; WBL, average wake bout length; RAR, rest/activity rhythm; IS, Standard RAR interdaily stability; IV, Standard RAR intradaily variability; RA, Standard RAR relative amplitude; fPC, functional principal component; Day, 24-hour interval. *p*-values are from independent-samples t-test or Wilcoxon rank-sum test for continuous variables and chi-square or Fisher exact test for categorical variables comparing the Normals vs. MCI; effect sizes represent the Cohen’s *d* estimate. *P*-values are from linear regression for sleep and circadian RAR parameters comparing the Normals vs. MCI, after adjustment for demographics and APOE genetic status; effect sizes represent the Eta-Squared estimate. Bold text indicates *p* < 0.05.

**Table 2.** Etiologic and syndromic diagnoses

Characteristic	*n* (%)
Etiology	
MCI due to Possible/Probable AD	24 (92.3)
+ Vascular Contributions	2 (7.7)
MCI due to Non-AD Etiology	2 (7.7)
+ Vascular Contributions	1 (3.8)
Syndromic Diagnosis	
Amnestic MCI	9 (34.6)
Non-Amnestic MCI	17 (65.4)

MCI, mild cognitive impairment; AD, Alzheimer’s disease. Individuals can be diagnosed with more than one etiology.

### Novel circadian RAR measures

**Figure 1.**
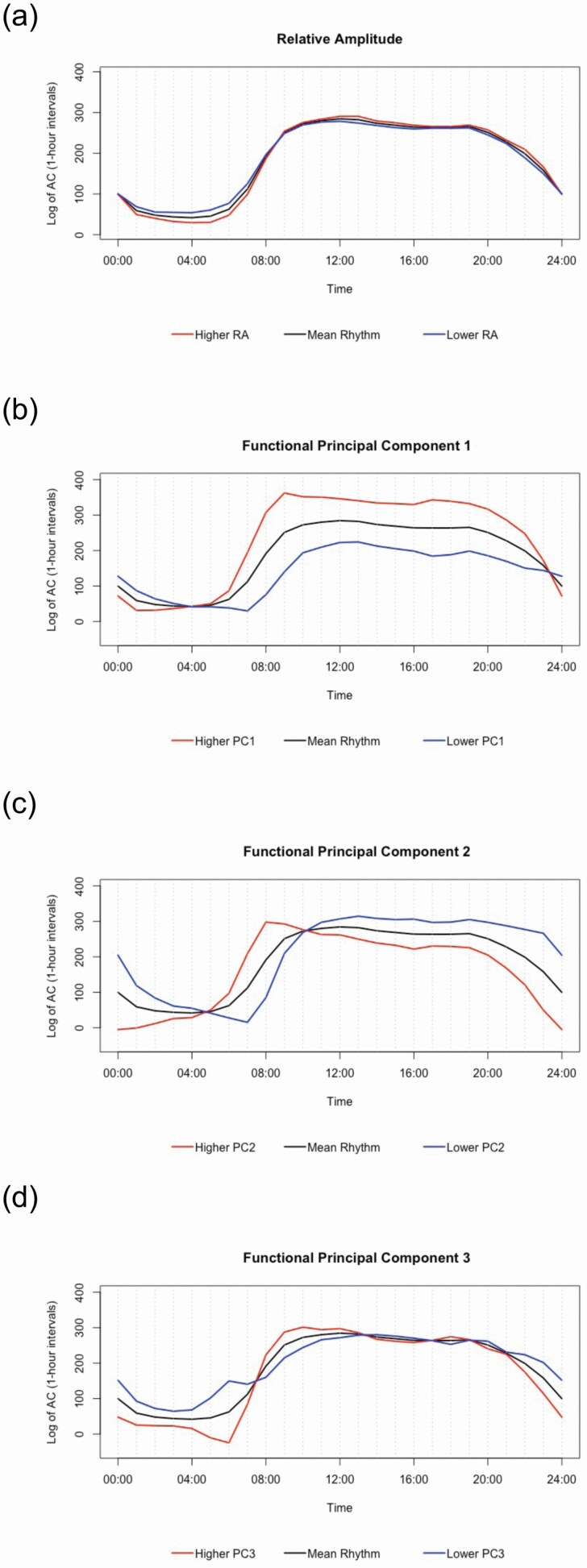
Visualization of Standard and Novel Circadian Rest/Activity Rhythms. *Panel (a)* represents the standard nonparametric rest/activity rhythm (RAR) parameter relative amplitude (RA). RA was dichotomized for visualization using a median split. The *black line* represents the mean rhythm of the overall sample, whereas the *red line* represents the mean rhythm among individuals with higher RA and the *blue line* reflects the mean rhythm among those with lower RA. *Panels* (b), (c), and (d) reflect the novel RAR parameters, functional principal components (fPC) 1, 2 and 3. In each panel, the *black line* represents the mean rhythm of the overall sample. The *red line* is two standard deviations above the mean for each respective fPC score, while the *blue line* is two standard deviations below the mean. Combined, fPC1, fPC2, and fPC3 account for approximately 69% of the total variation in RARs.

Three principal components were generated: fPC1, fPC2, and fPC3, which accounted for approximately 69% of the total variance. These functional principal components are displayed in Figure 1, b–d.

fPC1 appears to reflect the total volume of activity and the rhythm strength. Higher scores on fPC1 (*red line*), compared to the mean rhythm (*black line*), are characterized by a higher amplitude that begins to rise at 5 am, peaks at 9 am, and remains elevated until 11 pm, at which point higher scores drop below the mean rhythm and remain lower across the night (Figure 1b). fPC1 represents 33.5% of the total variance.

fPC2 appears to reflect the timing of the peak rhythm. Higher scores on fPC2 (*red line*), compared to the mean rhythm (*black line*), are characterized by an advanced timing of the rhythm peak that begins to rise at 4 am and peaks at 9 am. At 10 am, fPC2 scores drop below the mean rhythm and remain lower across the day and night (Figure 1c). fPC2 represents 26.7% of the total variance.

fPC3 appears to reflect the relative rhythm amplitude and the night-morning rhythm contrast. Higher scores on fPC3 (*red line*), compared to the mean rhythm (*black line*), are characterized by a higher amplitude that begins to rise at 6 AM and peaks at 10 AM. From 12 PM to 9 PM, fPC3 scores remain roughly level with the mean rhythm, after which they drop below the mean rhythm and remain lower across the night (Figure 1d). Note that lower levels of each fPC are plotted in *blue* in Figure 1, b–d. fPC3 represents 8.5% of the total variance. Details of the sleep and circadian RAR measures are summarized in Supplementary Table 1.

### Associations of diagnostic status with sleep and circadian RARs

**Figure 2.**
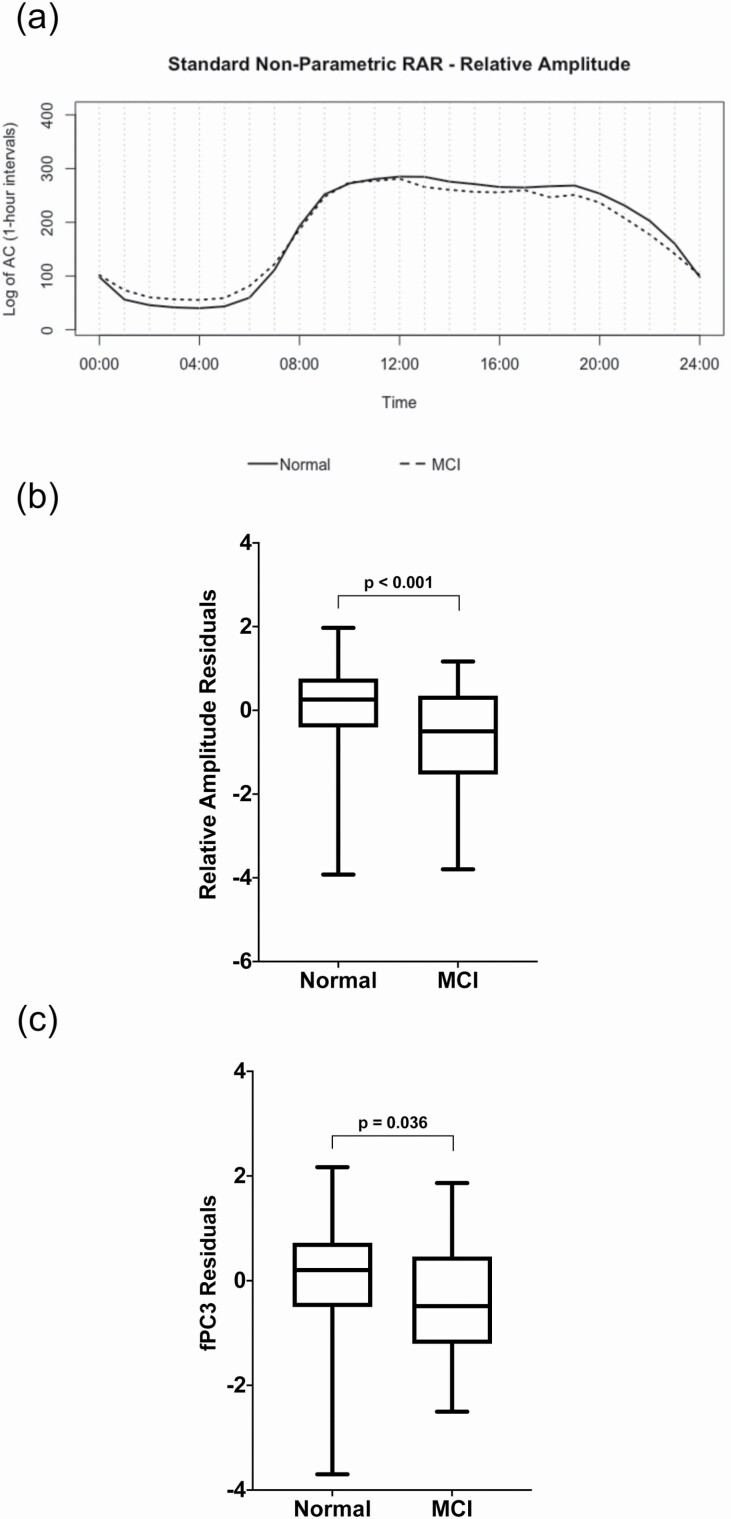
Standard and Novel Circadian Rest/Activity Rhythms in the Normals vs. MCI. Panel (a) represents the standard nonparametric rest/activity rhythm (RAR) parameter relative amplitude (RA) in the Normals vs. MCI. The *solid line* reflects the mean rhythm of the Normals, while the *dashed line* shows the mean rhythm of the MCI group. Panel (b) demonstrates that RA is significantly different between the Normals and MCI subjects. Panel (c) demonstrates that functional principal component 3 (fPC3) is significantly different between the Normals and MCI subjects. For panels (b) and (c), y-axis units represent the standardized residuals after adjustment for age, gender, education, and APOE ε4 genotype.

In the first set of regression analyses, several of the standard sleep and RAR measures were significantly different between the participants with a diagnosis of Normal compared to those with a diagnosis of MCI. After adjustment for age, gender, education, and APOE ε4 status, the MCI subjects had lower SE (β = −0.51, 95% confidence interval [CI] = −0.94, −0.09, *p* = 0.019) and lower RA (β= −0.78, 95% CI = −1.20, −0.37, *p* < 0.001) (Figure 2a). However, group differences in SE did not survive correction for multiple comparisons.

Comparisons involving the novel RAR measures indicated that the MCI subjects differed from the Normals on fPC3 (β = −0.46, 95% CI = −0.89, −0.03, *p* = 0.036; see Figure 2b). Diagnostic status was not associated with any other sleep or circadian RAR parameter (*p* ≥ 0.089).

### Associations of standard sleep parameters with cognitive composite scores

None of the standard sleep parameters were associated with executive function or episodic memory scores (*p* ≥ 0.082). Although regression analyses examining the association between the cognitive composite scores and the sleep parameters among all of the participants combined suggested that greater WASO was associated with a lower executive function scores (β = −0.15, 95% CI = −0.28, −0.01, *p* = 0.036), this finding did not survive correction for multiple comparisons.

### Associations of standard circadian RAR parameters with cognitive composite scores

**Figure 3.**
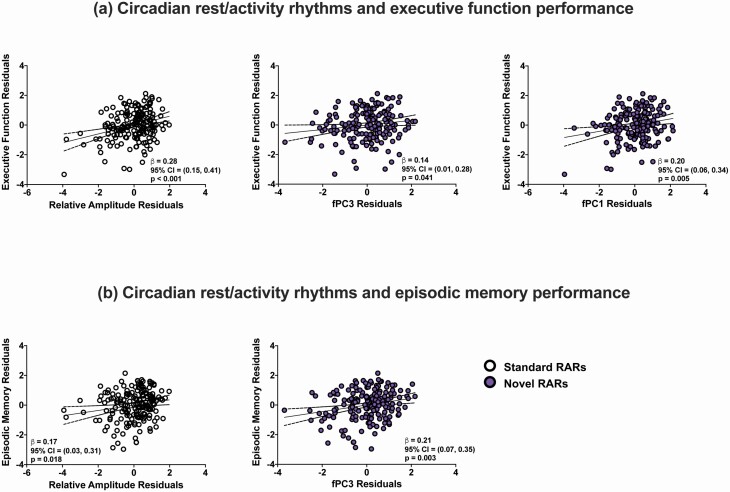
Associations of Standard and Novel Circadian Rest/Activity Rhythms with Cognitive Performance. Panel (a) demonstrates the associations of both the standard (*open circles*) and novel rest/activity rhythm (RAR) parameters (*purple dots*) with executive function performance. Panel (b) depicts the associations of both the standard and novel RAR parameters with episodic memory. Associations are depicted using partial regression plots of the standardized residuals after adjustment for age, gender, education, and APOE ε4 genotype.

In regression models examining the relationship between the standard nonparametric circadian indices and the cognitive composite scores, for the entire sample taken as a whole, higher IS (β = 0.15, 95% CI = 0.02, 0.29, *p* = 0.029), higher RA (β = 0.28, 95% CI = 0.15, 0.41, *p* < 0.001; Figure 3a), and lower IV (β = −0.15, 95% CI = −0.29, −0.01, *p* = 0.037) were associated with better executive function performance. Additionally, higher IS (β = 0.142, 95% CI = 0.003, 0.280, *p* = 0.045) and higher RA (β = 0.17, 95% CI = 0.03, 0.31, *p* = 0.018; Figure 3b) were associated with a higher episodic memory score. No relationship was found between IV and episodic memory (*p* = 0.187).

### Associations of novel circadian RAR parameters with cognitive composite scores

Regression models examining the relationship between the novel RAR parameters and cognitive composite scores demonstrated a significant association of higher fPC3 scores with higher executive function (β = 0.14, 95% CI = 0.01, 0.28, *p* = 0.041, Figure 3a) and episodic memory score (β = 0.21, 95% CI = 0.07, 0.35, *p* = 0.003). There was also a significant association between higher fPC1 and higher executive function scores (β = 0.20, 95% CI = 0.06, 0.034, *p* = 0.005, Figure 3b). fPC1 was not associated with episodic memory, and fPC2 was associated with neither executive function nor episodic memory scores (*p* > 0.183). The associations of sleep and circadian RAR parameters with cognition in each diagnostic group were not evaluated separately because we did not hypothesize differential relationships by diagnosis, and to constrain the total number of statistical comparisons.

### Correlations between the standard and novel actigraphy parameters

As depicted in Supplementary Figure 1, the novel RAR parameter fPC1 was correlated with all of the standard RAR parameters, which included a strong positive correlation with IS (*r* = 0.68, *p* < 0.001) and a strong negative correlation with IV (*r* = −0.68, *p* < 0.001). Similarly, fPC3 was correlated with almost all of the standard RAR parameters, which included weak-to-moderate positive correlations with RA (*r* = 0.42, *p* < 0.001) and IS (*r* = 0.29, *p* < 0.001), and a weak negative correlation with IV (*r* = −0.17, *p* < 0.019). The novel RAR parameters also shared weak-to-moderate correlations with many of the standard sleep parameters (Supplementary Figure 2). By design, the novel RAR parameters were not correlated with each other.

The first set of sensitivity analyses indicated that the relationships described above were not significantly altered by the inclusion of the interaction term for APOE ε4 status in the models (*p*-value for interaction term ≥ 0.238) (data not shown). The second set of sensitivity analyses demonstrated a similar pattern of results when the individuals with a diagnosis of “Impaired Not MCI” were excluded (*n* = 31), with the exception that the relationships between higher IS and better episodic memory performance (*p* = 0.085), and between higher RA and better episodic memory performance (*p* = 0.070) were attenuated.

## Discussion

This study demonstrates significant alterations in several aspects of circadian rhythms among individuals with MCI compared to the Normals. Specifically, MCI subjects had lower RA (a standard nonparametric RAR metric) compared to the Normals. Additionally, individuals with MCI were significantly different from the Normals on fPC3, a novel circadian RAR parameter, which reflected a lower rhythm peak as well as greater activity during the night and less activity in the morning compared to the mean values.

Primary analyses examining the associations of both standard and novel actigraphic parameters with cognitive performance in the combined sample demonstrated associations between several circadian RAR parameters and cognitive function. For example, there was a significant association between the novel RAR parameter fPC3 and both better executive function and episodic memory performance, while fPC1 was associated with better executive function performance. Additionally, there were associations between higher amplitude (RA) and greater rhythm consistency (IS) and both better executive function and episodic memory, whereas lower rhythm fragmentation (lower IV) was associated with better executive function. The size of the analytic sample precluded a more detailed evaluation of additional potential confounders, such as depressive symptoms and medication use.

The present study extends the literature on sleep, circadian rhythms, and AD by demonstrating significant differences between MCI subjects and normal controls using novel, actigraphy-derived, circadian RAR parameters. These findings demonstrated a difference in circadian rhythmicity that was not only present over the course of the entire night or daytime, as suggested by prior research, but rather reflects differences in rhythm amplitude and phase, and rest/activity changes during the night and early part of the day (as shown in fPC3). It is noteworthy that the Normals had both greater sleep efficiency and higher scores on fPC3, compared to the MCI subjects. fPC3 is characterized by a rhythm that is lower during the night and higher during the early part of the day, suggesting that fPC3 reflects both better sleep during the night, and greater arousal during the morning. Moreover, fPC3 levels were significantly lower among MCI subjects, compared to the Normals, and among the entire sample, fPC3 levels were related to both poorer executive function and episodic memory performance, as noted above. Coupled with the link between fPC1 (which reflects stronger rhythms) and executive function, our findings underscore the possibility that these pattern differences may, in part, underlie associations with cognition

Analyses examining the correlation of the novel fPCs with the standard RARs demonstrated that both fPC1 and fPC3 were highly correlated with nearly all of the standard circadian RAR parameters, suggesting that these novel measures highlight subtle, yet informative patterns in the actigraphy data that integrate specific circadian features represented by the standard metrics. Additionally, because the fPCs are not correlated with each other, they provide independent pieces of information that can be visualized over the 24-hour interval, illustrating a broader picture of circadian rhythm changes during the early phase of AD.

Interestingly, while fPC2 captures 26.7% of the variance in the actigraphy data, it was unrelated to both diagnostic status and cognition in this sample. The correlational analyses indicated that fPC2 was strongly associated with one of the parametric RAR measures – acrophase, and neither fPC2 nor acrophase were significantly different between the two diagnostic groups or related to cognitive performance (Supplementary Materials). Considering the differences in rhythm phase depicted in Figure 1c (illustrating fPC2), and the strong correlation between fPC2 and acrophase (timing of the rhythm peak); it is plausible that fPC2 most accurately reflects differences in the rhythm phase. Likewise, the weak, and mostly null correlations between fPC2 and the standard circadian parameters, suggest that fPC2 does not reflect the most dominant circadian patterns quantified by those standard measures.

The results of the present study build on prior literature in a number of ways. Prior studies, using standard nonparametric RAR measures, have reported altered RARs among older adults across the spectrum of AD. Overall, such work has shown that older adults exhibit reduced rhythm amplitude, and less stable, more fragmented rhythms, that become increasingly abnormal among those with MCI and after the onset of dementia [27]. These metrics do not, however, provide information about any shifts that may occur in the temporal details of the 24-hour circadian profile. The novel fPCA-derived circadian RARs described here appear to capture the same information as the standard RAR metrics, but also information about the shape and oscillatory pattern of rhythms over the 24-hour period, potentially providing a more complete picture of the participant’s circadian profile.

Our results demonstrating differences in circadian parameters between MCI subjects and the Normals at least partially confirm previous reports. For example, one such study [6] using both polysomnography and a measure of dim light melatonin onset revealed that MCI subjects had earlier onset of melatonin secretion, suggesting an advanced circadian phase and greater WASO, compared to normal controls. Our findings that sleep and circadian RARs are linked with cognitive performance are also similar with the results from three large cohort studies, which demonstrated that among older adults with varied cognitive status, greater actigraphy-measured sleep disturbance (WASO) and longer sleep duration are associated with worse global cognition and executive function performance [9, 10]; whereas more fragmented RARs are linked with worse executive function and visuospatial performance [11]. While these latter studies provide objective insights about sleep and circadian-related alterations among participants with a range of cognition and etiologies, the heterogeneous study populations with regard to cognitive abilities makes it difficult to ascertain when and to what degree these changes occurred in the context of AD. Additionally, several prospective studies have demonstrated that, among individuals with normal cognition at baseline, sleep and circadian rhythm measures are associated with an increased likelihood of progression to MCI or AD dementia [28–32].

The novel circadian RAR measures presented here may have particular utility in a clinical setting as they integrate a large amount of circadian information into a single, comprehensive profile, and thus may be useful in improving the diagnosis and treatment of specific circadian disorders (e.g. delayed/advanced sleep phase disorder, jet lag, shift work disorder). Additionally, these novel metrics may be useful in tracking the efficacy of medications and/or non-pharmacologic sleep and circadian interventions aimed at treating sleep disorders.

### Potential mechanisms

A number of factors may help explain the sleep and circadian differences observed between subjects with MCI and normal controls, and the relationships of these parameters with cognition. Experimental human and animal studies demonstrate that prolonged wakefulness, both during the day and after a night of sleep deprivation, increases neuronal excitability [33–35]. Evidence from animal models demonstrate that disproportionately higher levels of neuronal activity elevate extracellular brain β-amyloid [36, 37]—one of the hallmark features of AD. Conversely, research in both humans and animals indicate that sleep promotes large-scale synaptic downscaling [33, 38, 39] and may facilitate the clearance of neurotoxic waste (e.g. β-amyloid and tau) from the brain’s interstitial space—a process that may be altered in AD [40–45]. Good sleep promotes the removal of metabolic waste, which helps maintain neurovascular function (i.e. coordination and transfer between neurons and the brain’s microvasculature), and is associated with better cognition [46]. Other potential contributing factors linking disrupted sleep-wake patterns and cognition may pertain to trophic factors in the brain. For example, poor sleep quality and disrupted circadian rhythms, including insomnia, short sleep duration, and circadian phase shifts have been associated with altered levels of serum brain derived neurotrophic factor (BDNF) [47, 48]. BDNF plays a crucial role in synaptic plasticity and long-term potentiation, which facilitates learning and memory [49]. Thus, sleep and circadian-related changes in the brain’s neurotrophic signaling pathways—which regulate dendritic outgrowth, differentiation, and neuronal survival—may in part, underlie the changes in cognition that occur during the early phases of AD; highlighting the possibility that sleep and circadian rhythms could serve as modifiable targets for altering the progression of disease.

### Limitations and future opportunities

Although this study provides new information about the nature of sleep and circadian rhythms in the context of early AD, it is not without limitations. We relied on wrist actigraphy, which quantifies sleep and circadian RARs through motor activity rather than polysomnography—the gold standard sleep measure—or oscillations in core body temperature (or melatonin) over the 24-hour day that are closer to the endogenous or entrained circadian rhythm. While we adjusted for a number of covariates, this was an observational study, which by design inherently increases the risk of both confounding and selection bias. Additionally, because participants in this study were well educated, primarily white, and had a strong familial risk of AD dementia, the findings may not generalize to other older populations. Additionally, these were cross-sectional analyses including a number of well-characterized cognitively normal participants, and a more limited number of MCI subjects, which precluded our ability to examine the associations of alterations in sleep and circadian rhythms with the progression of disease, highlighting the need for future analyses to replicate these findings. A longitudinal study design would allow the implementation of more sophisticated statistical analysis techniques (analysis of longitudinal data, including moderation and/or mediation) to help identify between-group differences in sleep and circadian rhythms and their association with cognitive trajectories and likelihood of disease progression. Such analyses could help develop the case for the potential causal effects of poor sleep and altered circadian RARs on cognitive decline and AD clinical progression. Another key avenue for future research will be to evaluate the associations of sleep and circadian rhythms with patterns of brain connectivity—including in both structural and functional networks—to better understand the potential neural mechanisms underlying sleep and circadian-related cognitive changes in early AD.

## Conclusions

The current study provides evidence for sleep and circadian rhythm alterations in MCI, which are significantly associated with cognitive performance. Moreover, we demonstrate, using novel circadian RAR measures, a difference in circadian rhythm patterns that not only pertains to the nighttime, but—unlike nighttime sleep measures—extends into the day. As such, these novel metrics may provide additional insights into the relationship between RAR alterations and cognition during the early phase of AD, and may thereby improve the detection of subtle sleep and circadian rhythm changes in early AD. Future research is necessary to better understand both the longitudinal nature of these relationships and the potential neural mechanisms that underlie them. In particular, exploring how sleep and circadian RARs are related to patterns of structural and functional brain connectivity, and any potential moderating effects (such as those related to APOE ε4 status or brain β-amyloid burden) on these relationships will be important next steps.

## Supplementary Material

zpab007_suppl_Supplementary_Materials

## Data Availability

The data presented in this report are available upon request from any qualified investigator for the purposes of reanalysis and replication of results. See the BIOCARD website (www.biocard-se.org) for further details.
