# Supplementary material for: Associations of actigraphic sleep and circadian rest/activity rhythms with cognition in the early phase of Alzheimer’s disease
Source: Sleep Adv. 2021 Apr 27;2(1):zpab007. doi: 10.1093/sleepadvances/zpab007 (PMC8168567; doi:10.1093/sleepadvances/zpab007)
Supplement: zpab007_suppl_Supplementary_Materials [file zpab007_suppl_supplementary_materials.docx]

**Associations of Actigraphic Sleep and Circadian Rest/Activity Rhythms with Cognition in the Early Phase of Alzheimer’s Disease**

Alfonso Alfini^1^; Marilyn Albert^1^; Andreia V. Faria^2^; Anja Soldan^1^; Corinne Pettigrew^1^; Sarah Wanigatunga^4^; Vadim Zipunnikov^3^; Adam P. Spira^4^

^1^Department of Neurology, Johns Hopkins School of Medicine, Baltimore, MD, USA^. 2^Department of Radiology and Radiological Science, Johns Hopkins School of Medicine, Baltimore, MD, USA^. 3^Department of Biostatistics, Johns Hopkins Bloomberg School of Public Health, Baltimore, MD, USA^. 4^Department of Mental Health, Johns Hopkins Bloomberg School of Public Health, Baltimore, MD, USA

**Please Address Manuscript Correspondence to:**

Alfonso Alfini, PhD

1620 McElderry Street, Reed Hall 1 West, Baltimore, MD 21205 USA

Email: [aalfini@jhu.edu](mailto:aalfini@jhu.edu) | Telephone: +1 (970) 319-1616

**SUPPLEMENTAL MATERIALS**

| **Supplemental Table 1. Sleep and circadian rest/activity rhythm (RAR) parameters.** | |
| --- | --- |
| **Measure** | **Definition** |
| *Standard Sleep Parameters* |  |
| Total Sleep Time (TST) | Number of minutes slept while in bed. |
| Sleep Efficiency (SE) | Percentage of time in bed asleep. |
| Wake After Sleep Onset (WASO) | Number of minutes awake after initial sleep bout. |
| Average Wake Bout Length (WBL) | Number of minutes awake divided by the number of wake bouts. |
|  |  |
|  |  |
| *Standard Non-Parametric RAR Parameters* | |
| Interdaily Stability (IS) | Rhythm consistency across 24-hour intervals. |
| Intradaily Variability (IV) | Rhythm fragmentation within each 24-hour interval, averaged across intervals. |
| Relative Amplitude (RA) | Rhythm height as the difference between the most active 10 hours and least activity 5 hours of the 24-hour interval, averaged across intervals. |
|  |  |
| *Novel RAR Parameters* | |
| Functional Principal Component 1 (fPC1) | fPCs are defined by their shape and relative distance from the mean rest/activity rhythm, and reflect the dominant circadian pattern contrasts across the 24-hour interval. |
| Functional Principal Component 2 (fPC2) |  |
| Functional Principal Component 3 (fPC3) |  |

| **Supplemental Table 2. Standard parametric circadian rest/activity rhythm (RAR) parameters by clinical diagnosis.** | | | | |
| --- | --- | --- | --- | --- |
| Parametric RAR Measures | Total Sample (*n*=179) | Normal (*n*=153) | MCI (*n*=26) | *p* |
| Cosinor Amplitude, mean±SD, score | 2.1±0.4 | 2.2±0.4 | 2.0±0.4 | 0.089 |
| Cosinor MESOR, mean±SD, score | 3.1±0.5 | 3.1±0.5 | 3.1±0.4 | 0.652 |
| Cosinor Acrophase, mean±SD, score | -3.9±0.3 | -4.0±0.3 | -3.9±0.3 | 0.092 |

*Note.* SD - standard deviation; MESOR – Midline Estimating Statistic of Rhythm, amplitude – rhythm height from nadir to the rhythm peak, acrophase – timing of the rhythm peak, RAR – rest/activity rhythm.

| **Supplemental Table 3. Associations of standard parametric rest/activity rhythm (RAR) measures with executive function and episodic memory performance.** | | | |  |
| --- | --- | --- | --- | --- |
|  |  |  | **Cognitive Performance** |  |
| **Measure** | | | **Beta Coefficient (95% Confidence Interval)** | |
|  |  |  |  |  |
|  | |  | (*n*=179) |  |
| *Standard Parametric RAR Parameter* | |  | *Executive Function Composite* |  |
| Cosinor Amplitude | |  | **0.21 (0.08, 0.35)**** |  |
| Cosinor MESOR | |  | **0.14 (0.00, 0.29)*** |  |
| Cosinor Acrophase | |  | 0.05 (-0.08, 0.19) |  |
|  | |  |  |  |
|  | |  | *Episodic Memory Composite* |  |
| Cosinor Amplitude | |  | **0.15 (0.01, 0.29)*** |  |
| Cosinor MESOR | |  | 0.05 (-0.10, 0.19) |  |
| Cosinor Acrophase | |  | -0.04 (-0.18, 0.10) |  |
| *Note*. Beta coefficients and 95% confidence intervals are from linear regression examining the associations of the standard parametric rest/activity rhythm (RAR) parameters with executive function and episodic memory performance, after adjustment for demographics and APOE ε4 genetic status. Significant results are bolded and indicated by the following: * *p* < 0.05, ** *p* < 0.01, *** *p* < 0.00. | | | |  |

**
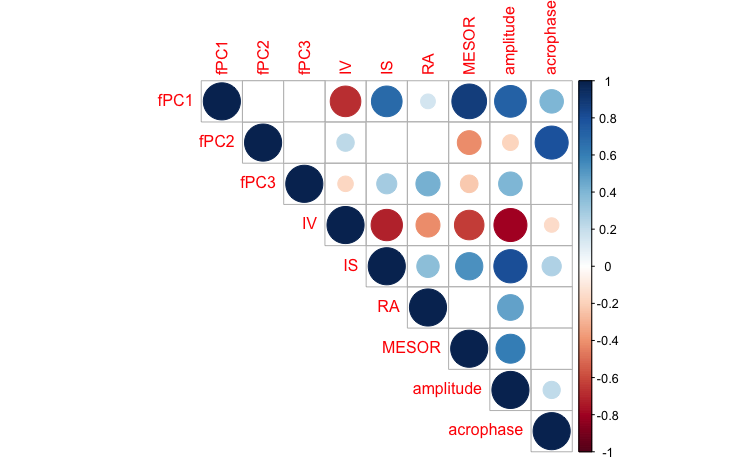
Supplemental Figure 1**

*Figure Title:* Standard and Novel Rest/Activity Rhythm Measures Intercorrelation Matrix

*Figure Caption:* Overall, standard non-parametric (IS, IV, and RA) and parametric (cosinor) RAR metrics (MESOR, amplitude, and acrophase) are moderately correlated with each other. Standard parametric and non-parametric metrics are moderately correlated with fPC1, but most correlations diminish after fPC1. Functional principal components are not correlated with each other, by design.

**
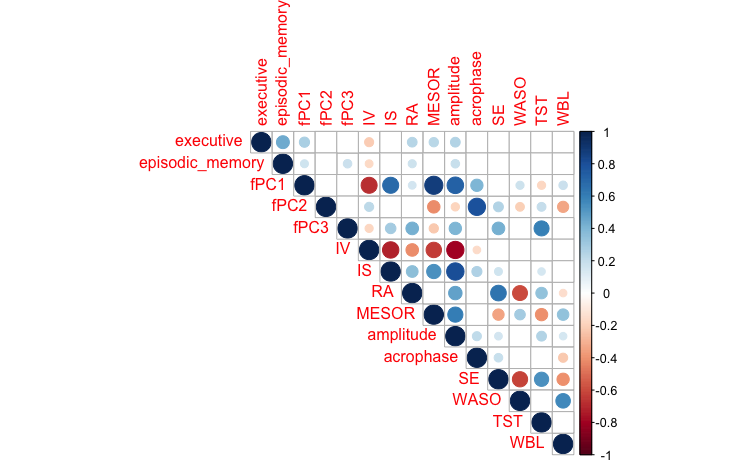
Supplemental Figure 2**

*Figure Title:* Standard and Novel Actigraphy Measures Intercorrelation Matrix with Cognitive Composites

*Figure Caption:* Standard parametric (cosinor) RAR metric amplitude is associated with executive function and episodic memory, and both amplitude and MESOR are associated with executive function. Standard non-parametric measures IV and RA are associated with executive function and episodic memory. The novel RAR metric principal component 1 (fPC1) is associated with both episodic memory and executive function and novel RAR fPC3 is associated with episodic memory.

***Results from Supplemental Analyses of Parametric RAR Measures***

Supplemental analyses compared the circadian RAR measures derived using the standard parametric (cosinor) approach. The results comparing the controls and MCI subjects, showed no significant differences by diagnostic status (*p* ≥ 0.089). Adjusted models assessing the relationship between the standard parametric RAR measures and the cognitive composite scores demonstrated that both greater rhythm amplitude (β = 0.21, 95% CI = 0.08, 0.35, *p* = 0.003) and a higher MESOR (β = 0.14, 95% CI = 0.00, 0.29 , *p* = 0.049) were associated with a better performance on executive function tests. Additionally, greater amplitude was associated with a higher episodic memory score (β = 0.15, 95% CI = 0.01, 0.29, *p* = 0.039). Acrophase was not significantly associated with executive function, and neither MESOR nor acrophase were related to episodic memory scores (*p* ≥ 0.442) (See **Supplemental Table 3**).
